# Modulation of Heat Shock Protein Expression in Alveolar Adenocarcinoma Cells through Gold Nanoparticles and Cisplatin Treatment

**DOI:** 10.3390/pharmaceutics16030380

**Published:** 2024-03-11

**Authors:** Bashiru Ibrahim, Taiwo Hassan Akere, Swaroop Chakraborty, Eugenia Valsami-Jones, Hanene Ali-Boucetta

**Affiliations:** 1 Nanomedicine, Drug Delivery & Nanotoxicology (NDDN) Lab, School of Pharmacy, College of Medical and Dental Sciences, University of Birmingham, Birmingham B15 2TT, UK; 2 School of Geography, Earth and Environmental Sciences, College of Life and Environmental Sciences, University of Birmingham, Birmingham B15 2TT, UK

**Keywords:** cytotoxicity, apoptosis, oxidative stress, gold nanoparticles, cisplatin

## Abstract

Heat-shock proteins (HSPs) are stress-responsive molecules belonging to the family of evolutionary molecular chaperones known to be crucial in many cancer types, including human alveolar adenocarcinoma cells (A549). These proteins are highly overexpressed in cancers to support their ability to accommodate imbalances in cell signalling, DNA alterations, proteins, and energy metabolism associated with oncogenesis. The current study evaluated the effects of gold nanoparticles (AuNPs) combined with cisplatin (CDDP) on molecular chaperone HSPs in A549 cells. It was found that AuNPs:CDDP decreased the percentage of cell viability (38.5%) measured using the modified lactated dehydrogenase (mLDH) and 3-[4,5-dimethylthiazole-2-yl]-2,5-diphenyltetrazolium bromide (MTT) assays. AuNPs:CDDP exposure caused a significant (*p* < 0.05) increase in reactive oxygen species (ROS) generation by 1.81-fold, apoptosis induction, and a decrease in the mitochondrial membrane potential (MMP) compared to AuNPs or CDDP alone. Similarly, exposure to the AuNPs:CDDP combination had pronounced cytotoxic effects on the expression of HSPs and PI3K/AKT/mTOR, as well as apoptosis-related proteins. The results demonstrate that the combination of AuNPs with CDDP might enhance the anticancer efficacy of CDDP.

## 1. Introduction

CDDP is a well-known chemotherapeutic drug that is used to inhibit the growth of numerous human cancers, including A549 [1]. It depletes the growth of tumour cells by interfering with the complementary bases of DNA, leading to DNA damage, disabling repair mechanisms, and subsequently, resulting in cell death [2,3]. However, it is well-known that CDDP can potentially induce severe side effects in cancer patients, including kidney failure, immunity infections, irreversible hearing loss, and renal and neural damages. These side effects limit its use in anticancer therapy [2,4,5]. Additionally, tumour cells can promote resistance to CDDP by reducing its accumulation, thereby further limiting its application as an anticancer therapeutic in those patients [6,7,8]. To overcome these challenges, combination therapy with other drugs or nanoparticles (NPs) has been extensively considered to suppress drug resistance, decrease cancer cell viability, and enhance CDDP delivery to targeted tissues [9,10]. Among several NPs, AuNPs are widely used to tackle these challenges by capitalising on properties such as size tunability, large surface area-to-volume ratio, optical effects, ease of functionalisation, and biocompatibility [11]. Studies reported that AuNPs can contribute towards overcoming drug resistance by delivering the drug to a target site, where activating the ability to surpass resistance mechanisms is possible [12,13]. Examples include the combination of CDDP with AuNPs, which was reported to selectively decrease the proliferation of A549, human osteosarcoma (MG-63), and sarcoma osteogenic (Saos-2) cells through ROS generation [14]. As a further example, AuNPs conjugated with CDDP were tested in A549 cells in vitro. The study reported that AuNPs:CDDP results in greater efficacy in cancer therapy compared to the free drug alone [15]. Similarly, the co-administration of CDDP-conjugated PEG-AuNPs was found to induce the cytotoxicity of A549 cells by overcoming CDDP resistance [16]. However, the precise molecular effects of AuNPs and CDDP combination against HSPs in A549 were never investigated.

HSPs belong to the family of evolutionary molecular chaperones and are responsible for signal transduction, cell cycle regulation, cell death, cell development, and maintaining cell survival [17,18]. The HSPs are classified, based on their molecular weight, into three main categories: small HSPs (HSP27); HSP40, the chaperone family of HSP60, HSP70, HSP90; and the large HSPs (HSP110, glucose-regulated proteins 170 GRP170) [19,20]. Notably, because of the exposure of cells to environmental conditions such as biochemical agents, ultraviolet light, hyperthermia, low oxygen levels, toxicants, and other homeostatic stressors, the proteins are promptly elevated to augment the stress factor referred to as the heat shock response (HSR), which is controlled predominantly by the heat shock factor (HSF) [19,21]. When HSF is phosphorylated, it rapidly binds to the heat shock element (HSE) in the nucleus and accelerates the overexpression of HSPs [22,23].

Under “normal’’ conditions, increased HSPs, in response to stress, provide cytoprotective functions to normal cells. However, the dysfunction of these proteins contributes to the development of several disorders and diseases, such as cancer [24,25,26]. In cancer, the HSPs are highly overexpressed in response to metabolic needs such as hypoxia, acidosis, and nutrient-deprived tumour conditions [27,28,29]. The proliferation of cancer occurs through the activation of oncogene proteins and the amplification and elevation of intracellular signalling proteins that require the overexpression of HSPs for their stabilisation, ubiquitination, protein folding, aggregation, and proteolytic degradation [25,30,31,32]. Thus, the inhibition of molecular HSPs may offer a novel way to prevent cancer proliferation, and targeting multiple HSPs may also provide a new direction in cancer treatment with the development of novel therapeutic modalities.

Herein, it was hypothesised that a combination of AuNPs with CDDP could downregulate the overexpression of HSPs and the PI3K/AKT/mTOR pathway and upregulate the expression of apoptosis-related proteins. Firstly, the cytotoxicity of AuNPs and CDDP alone was evaluated in A549 cells. Secondly, the cell viability of A549 following the combination of AuNPs with CDDP was studied. Thirdly, the underlying mechanisms of cytotoxicity, including oxidative stress, MMP, and apoptosis caused by AuNPs:CDDP, were investigated. Finally, the effects of the AuNPs:CDDP combination HSPs, PI3K/AKT/mTOR signalling, and apoptotic proteins in A549 cells were evaluated.

## 2. Materials and Methods

### 2.1. Materials and Reagents

The 10 nm AuNPs coated with citrate used for this study were purchased from NanoComposix (San Diego, CA, USA). Phosphate-buffered saline (PBS), F12 nutrimix media, penicillin/streptomycin, 0.25% trypsin–EDTA, foetal bovine serum (FBS), MitoHealth, Alexa fluor 488 Annexin V/PI, and RPMI 1640 were purchased from Thermo Fisher Scientific (Paisley, UK). CytoTox 96 non-radioactive cytotoxicity, caspase-Glo 3/7, and GSH/GSSG-Glo luminescence assay kits were acquired from Promega (Southampton, UK). CDDP, Menadione, Staurosporine, dimethyl sulfoxide (DMSO), tris-base, glycine, MTT (3-(4,5-dimethylthiazol-2-yl)-2,5-diphenyl-2H-tetrazolium bromide), 2,7-dichlorofluorescin diacetate (DCFDA), and sodium chloride were purchased from Sigma Aldrich (Dorset, UK). Bovine serum albumin (BSA), acrylamide/bis-acrylamide, and ponceau were bought from Alfa Aesar (Lancashire, UK). The PVDF membrane and ECL were purchased from GE HealthCare (Buckinghamshire, UK). HSP40 (C64B4), HSP60 (D6F1), HSP70 (D1M6), HSP90 (C45G5), AKT (C67E7), PTEN (138G6), mTOR (7C10), GSK-3β (27C10), GAPDH (D16H11), MDM2 (D1V2Z), caspase-3 (D3R6Y), caspase-9 (C9), and PARP (46D11) primary antibodies were purchased from Cell Signaling Technology (CST) (London, UK).

### 2.2. Cell Culture of A549 Cells

A549 was obtained from the American Type Culture Collection (ATCC) and grown in F-12 Ham media with FBS (10%) and 100 µg/mL antibiotics (penicillin/streptomycin (1%)) at 37 °C in a 5% CO_2_. The cells were passaged after reaching 70-80% confluency. AuNPs, CDDP, and AuNPs:CDDP were diluted in F-12 Ham media with respect to the concentration of the experiments and vortexed for 10s before being added to cells.

### 2.3. Cytotoxicity Assays

MTT assay was used to investigate the percentage cell viability of the cells. In brief, A549 cells were seeded on 96-well plates at a density of 7000 cells/well overnight and treated with 10 nm AuNPs at concentrations ranging from 10 to 45 µg/mL, CDDP (1.25–10 µg/mL), and AuNPs:CDDP. Control cells were incubated with complete culture media (CCM) containing FBS (10%) and 100 µg/mL antibiotics (penicillin/streptomycin (1%)) while the positive control cells were incubated with 20% DMSO for 24 and 48 h. After the exposure time, the medium was removed from each well and replaced with 120 µL of MTT (5 mg/mL prepared in PBS), followed by incubation for 4 h at 37 °C. A total of 200 µL of DMSO was added into each well to solubilise the formazan crystals formed by the cells. The absorbance was measured with a FLUOstar Omega microplate reader (BMG LABTECH Ltd., Aylesbury, UK) at 570 nm. For the mLDH, the cells were lysed with Triton X100 (0.9%) and incubated at 37 °C for 1h. The lysates were collected in Eppendorf tubes and centrifuged for 5 min at 4 °C. A total of 50 µL of the supernatants were transferred to a new 96-well plate, and 50 µL of substrate mixture was added, followed by incubation for 15 min at room temperature covered with foil. After the incubation, 50 µL of stop solution was added, and the absorbance of each sample was measured using a FLUOstar Omega microplate reader (BMG LABTECH Ltd., Aylesbury, UK) at 490 nm.

### 2.4. Measurement of Oxidative Stress Parameters

ROS generation levels in A549 cells treated with AuNPs (25 µg/mL), CDDP (2.191 µg/mL), and AuNPs:CDDP were measured using 2,7-Dichlorofluorescin DCFDA as described [33]. Intracellular glutathione was determined using GSH/GSSG-Glo assay kit according to the manufacturer’s instructions (Promega, Southampton, UK). In brief, A549 cells were seeded on a 96-opaque-well plate at a density of 7000 cells/well overnight and then incubated with AuNPs (25 µg/mL), CDDP (2.191 µg/mL), and AuNPs:CDDP for 24 h at 37 °C. Subsequently, the cells were washed with 1% PBS and lysed with 50 µL of either total glutathione or oxidised glutathione reagent. A total of 50 µL of luciferin generation reagent was added to all the wells and incubated for 30 min at room temperature. Luciferin reagent (100 µL) was added to each well and incubated at room temperature for 15 min. The luminescence intensity was measured using a FLUOstar Omega microplate reader (BMG LABTECH Ltd., Aylesbury, UK).

### 2.5. Mitochondrial Membrane Potential (MMP)

MMP changes induced after treatment were evaluated according to the method described by [34]. In brief, A549 cells were seeded on a 96-well plate at a density of 7000 cells/well overnight and treated with AuNPs (25 µg/mL), CDDP (2.191 µg/mL), and AuNPs:CDDP for 24 h. After the treatment, 50 µL of mitochondrial staining solution was added to each well and incubated for 30 min at 37 °C. The cells were then counterstained with 100 µL of Hoechst 33342 staining solution and incubated for another 15 min at room temperature. The cells were then washed with 100 µL of PBS three times, and a further 200 µL of PBS was added to each well. The fluorescence intensity was captured using a FLUOstar Omega microplate reader (BMG LABTECH Ltd., Aylesbury, UK) in a time-resolved fluorescence manner, and the quality of fluorescence intensity was captured using EVOS microscopy equipped with an appropriate filter for DAPI and GFP at 20× magnification.

### 2.6. Apoptosis Analysis

Apoptosis was evaluated using the Annexin V-FITC/PI (Thermo Fisher Scientific, Paisley, UK) and caspase-Glo 3/7 activity assay kits (Promega, Southampton, UK) according to the manufacturer’s instructions. For Annexin V-FITC/PI, A549 cells were seeded in six-well plates overnight at a density of 2 × 10^5^ cells/well and treated with AuNPs (25 µg/mL), CDDP (2.191 µg/mL), and AuNPs:CDDP for 24 h. Moreover, 10% DMSO and 1 µM Staurosporine were used as inducers of necrosis and apoptosis, respectively. The cells were detached and centrifuged at 1500 rpm for 5 min at 4 °C. The pellet was resuspended with 100 µL of Annexin binding buffer and incubated with 5 µL of Annexin V-FITC and 1 µL of PI for 15 min in the dark at room temperature. After the incubation, the cell suspension was further diluted with 400 µL of Annexin binding buffer and analysed by BD LSRFortessa™ X-20 flow cytometry (BD Biosciences, Franklin Lakes, NJ, USA) by acquiring at least 10,000 events.

For caspase-Glo 3/7 activity, A549 cells were seeded at a density of 7000 cells/well in 96-opaque well plates and allowed to attach for 24 h. The cells were treated with AuNPs (25 µg/mL), CDDP (2.191 µg/mL), and AuNPs:CDDP. After the treatment time point, the cells were additionally allowed to equilibrate for 1 h at room temperature. The media containing the treatments was gently removed and replaced with 100 µL of caspase-Glo 3/7 reagent. The contents were gently mixed for 60 s using a plate shaker at 445 g and then incubated at room temperature in a light-protected area. The luminescence was finally read using a FLUOstar Omega microplate reader (BMG LABTECH Ltd., Aylesbury, UK).

### 2.7. Western Blotting

For Western blotting analysis, cells were seeded on six-well plates at a density of 2 × 10^5^ cells/well overnight and treated with AuNPs (25 µg/mL), CDDP (2.191 µg/mL), and AuNPs:CDDP. After the treatment, cells were lysed with NP40 lysis buffer (containing mercaptoethanol and phenylmethylsulfonyl fluoride (PMSF)). The concentration of protein in the samples was determined using a Bradford assay kit (Dorset, UK). Specifically, a total of 20 µg of protein lysate were loaded to SDS-PAGE (Bio-Rad, Ltd., Watford, UK) and separated based on their molecular weight. The proteins were then transferred to a PVDF membrane (GE Healthcare, UK) in a running buffer prepared from NaCl, Tris-Base, and 20% methanol connected to a power supply with an accelerating voltage of 85V for 1:45 h. The membrane was blocked for 1 h in TBST with 5% non-fat milk at room temperature and incubated with primary antibodies HSP40, HSP60, HSP70, HSP90, AKT, PTEN, mTOR, GSK-3β, caspase-3, caspase-9, and GAPDH diluted in TBST with 5% BSA (1:1000) overnight at 4 °C on a shaker with gentle agitation. Following this, the membrane was washed with TBST three times and subsequently incubated with secondary antibodies (horseradish peroxidase-conjugated anti-rabbit or anti-mouse) diluted in TBST with 5% BSA (1:2000) at room temperature for 1 h. ECL reagent was then added to the membrane after washing three times. Finally, the protein bands were detected using G: BOX Chem XX6/XX9 (Syngene, Cambridge, UK).

### 2.8. Statistical Analysis

The experiments were repeated at least three times, and the data were expressed as the mean ± standard deviation (SD) for all the independent experiments. Data were analysed by one-way analysis of variance (ANOVA) followed by Bonferroni post hoc test for multiple comparisons to determine the statistical difference between control and treated cells using GraphPad Prism software (version 9.0. BD).

## 3. Results

### 3.1. Individual Cytotoxicity Assessments of CDDP and AuNPs 

**Figure 1:**
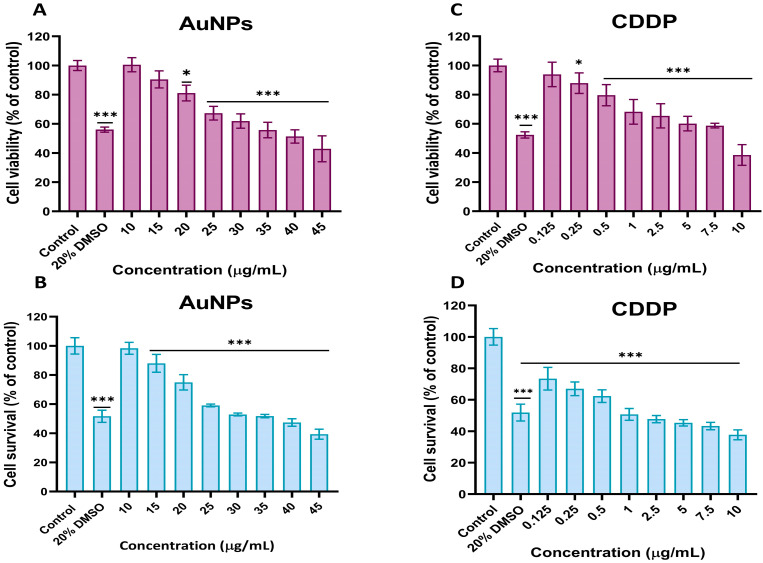
Percentage cell viability of A549 cells following treatment with different concentrations of AuNPs (0–45 µg/mL) or CDDP (0–10 µg/mL) for 24 h measured by MTT and mLDH assays. (**A**) MTT assay of AuNPs, (**B**) mLDH assay of AuNPs, (**C**) MTT assay of CDDP, (**D**) mLDH assay of CDDP. Additionally, 20% DMSO was used as a positive control. The plotted graphs represent the means ± standard deviation (SD) of six independent experiments. Bars with an asterisk (*) show statistical differences (* *p* < 0.05, and *** *p* < 0.001) compared with the control.

A549 cell responses to CDDP and AuNPs, individually, were assessed using the MTT and mLDH assays. A dose-dependent decrease in the percentage cell viability following treatment with AuNPs and CDDP alone for 24 and 48 h was observed with both assays (Figure 1). As shown in Figure 1A, exposure to AuNPs caused minimal cell toxicity at the tested concentrations (0–45 µg/mL) with maximum cell viability (43%) at the highest concentration (45 µg/mL) after 24 h. As expected, exposure to CDDP alone showed a significant reduction in cell viability up to 38.9% after 24 h at the highest concentration tested (10 µg/mL) (Figure 1B). In addition, A549 cell survival was measured using the mLDH assay, which indirectly measures the number of viable cells that remain after exposure to AuNPs or CDDP. mLDH was used because it is the most reliable cytotoxicity assay as it interferes less with NMs [35]. As shown in Figure 1C,D, AuNPs exhibit a reduced impact (IC_50_ = 43.01 µg/mL) on A549 cells compared to CDDP (IC_50_ = 2.191 µg/mL).

### 3.2. The Effects of AuNPs:CDDP on Cell Viability

**Figure 2:**
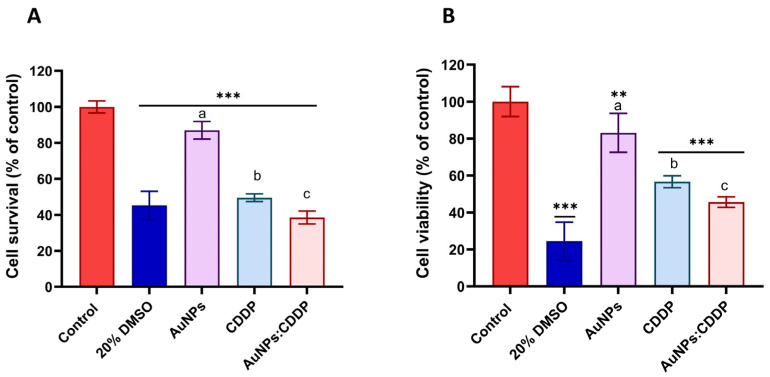
Percentage cell viability of A549 cells following treatment with a combination of AuNPs (25 µg/mL) and CDDP (2.191 µg/mL) for 24 h measured by mLDH and MTT assays. (**A**) mLDH assay of AuNPs, CDDP, and AuNPs:CDDP; (**B**) MTT assay of AuNPs, CDDP, and AuNPs:CDDP. Also, 20% DMSO was used as a positive control. The plotted graphs represent the means ± SD of three independent experiments. Bars with an asterisk (*) show statistical differences (** *p* < 0.01, *** *p* < 0.001) compared with the control. Data with different letters above the bars represent the statistically significant difference (*p* < 0.05) between the AuNPs:CDDP, CDDP, and AuNPs alone.

Based on viability data, the IC_50_ of CDDP (2.191 µg/mL) and 25 µg/mL of AuNPs were used to investigate the effects of the combination of AuNPs with CDDP. As shown in Figure 2A, the combination of AuNPs:CDDP significantly (*p* < 0.05) reduced the percentage cell survival to (38.5%) compared to AuNPs (87%) and CDDP alone (49.5%) as assessed by the mLDH assay. The cell viability following treatment with the combination of AuNPs:CDDP was further examined using the MTT assay. It was also found that the combination of AuNPs:CDDP significantly (*p* < 0.05) decreased the percentage of cell viability to (47%) compared to AuNPs (83%) and CDDP (57%) alone (Figure 2B). These results indicate that combinations of AuNPs:CDDP can enhance the reduction of A549 cell proliferation.

### 3.3. Effects of AuNPs:CDDP on Oxidative Stress Parameters

**Figure 3:**
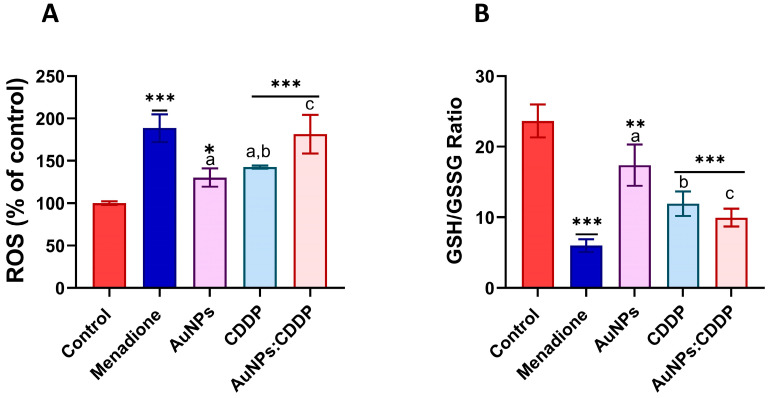
Effects of AuNPs, CDDP, and AuNPs:CDDP on oxidative stress biomarkers in A549 cells after 24 h. (**A**) ROS generation, (**B**) GSH/GSSG ratio. Menadione (50 µM) was used as a positive control. The plotted graphs represent the means ± SD of three independent experiments. Bars with an asterisk (*) show statistical differences (* *p* < 0.05, ** *p* < 0.01, and *** *p* < 0.001) compared with the control. Data with different letters above the bars represent the statistically significant differences (*p* < 0.05) between the AuNPs:CDDP, CDDP, and AuNPs alone.

Exposure to anticancer drugs and NPs can cause the formation of free radicals in the mitochondria [36]. Abnormal concentration of free radicals can potentially interfere with the function of the mitochondria, resulting in oxidative stress that damages cell membranes, DNA and inhibits the activation of certain signalling pathways such as P13/AKT/mTOR, MAPK/ERK, and Wnt [37]. The combined effect of AuNPs:CDDP on oxidative stress was determined by measuring the levels of ROS and intracellular glutathione in A549 cells. As can be seen in Figure 3A, the combination of AuNPs:CDDP increased the levels of ROS significantly (*p* < 0.05) compared to CDDP or AuNPs alone and control. ROS generation causes an imbalance in endogenous antioxidants such as glutathione that neutralise their oxidative damage [38]. An imbalance in intracellular glutathione homeostasis leads to the progression and development of many diseases, including cancer [39]. The results in Figure 3B show that the AuNPs:CDDP combination significantly (*p* < 0.05) reduced the ratio of GSH/GSSG to 9.95% compared to CDDP (12%), AuNPs (17.39%), and control (23.65%).

### 3.4. Effects of AuNPs:CDDP on Mitochondrial Membrane Potential (MMP)

**Figure 4:**
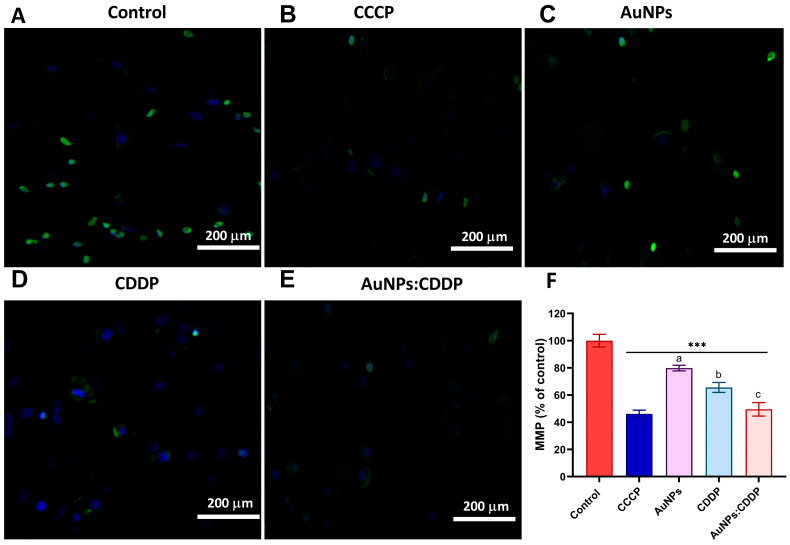
MMP of A549 cells following treatment for 24 h with (**A**) Control (**B**) 50 mM of CCCP, (**C**) AuNPs, (**D**) CDDP, (**E**) AuNPs:CDDP combination, (**F**) normalised fluorescence intensity. The plotted graphs represent the means ± SD of three independent experiments. Bars with an asterisk (*) show statistical difference (*** *p* < 0.001) compared with the control. Data with different letters above the bars represent the statistically significant difference (*p* < 0.05) between the AuNPs:CDDP, CDDP, and AuNPs alone.

The MMP of A549 cells treated with CDDP, AuNPs, and AuNPs:CDDP was measured using an HCS mitochondrial health stain kit. Carbonyl cyanid-3-chlorophynylhydrazone (CCCP) was used as a positive control. The MitoHealth stain signals change by accumulating in healthy mitochondria, producing higher fluorescence intensity proportional to MMP. Therefore, as the mitochondrial membrane is compromised, low fluorescence intensity is observed. As shown in Figure 4A–E, A549 cells treated with AuNPs, CDDP, and the combination AuNPs:CDDP show a lower fluorescence intensity compared to the control, suggesting a loss of MMP. Specifically, when the fluorescence intensity was normalised against the control, the AuNPs:CDDP combination displayed a statistically significant (*p* < 0.05) decrease in normalised fluorescence intensity value (49.47%) for MMP compared to AuNPs alone (78.84%) and CDDP alone (65.56%) as shown in Figure 4F. On the other hand, cells treated with 50 mM of CCCP exhibited a significant detriment on mitochondrial health, as demonstrated by its lowest normalised fluorescence intensity value (46.18%) for MMP. This shows that the CCCP induced mitochondrial disruption similar to AuNPs:CDDP treated cells.

### 3.5. Effects of AuNPs:CDDP on HSPs

**Figure 5:**
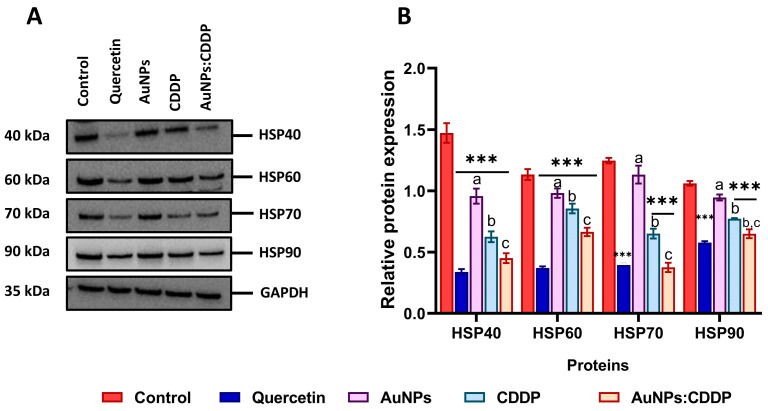
Effects of AuNPs, CDDP, and AuNPs:CDDP combination on the expression of HSPs (**A**) protein expression of HSP40, HSP60, HSP70, and HSP90 detected by Western blotting, (**B**) relative protein expression of HSP40, HSP60, HSP70, and HSP90 semi-quantified by ImageJ Software (Version 2.0.0, Public domain, BSD-2). Quercetin (10 µg/mL) was used as a positive control. The plotted graphs represent the means ± SD of three independent experiments. Bars with an asterisk (*) show statistical difference (*** *p* < 0.001) compared to control. Data with different letters above the bars represent the statistically significant difference (*p* < 0.05) between the AuNPs:CDDP, CDDP, and AuNPs alone.

The expression of HSPs is involved in the control of cell survival, metastasis, resistance to therapy, and avoidance of apoptotic cell death in many cancers, including A549 cells. It is, therefore, important to understand the effects of our combination therapy, as targeting these proteins might be an effective strategy in cancer therapy [24]. A549 cells were exposed to AuNPs, CDDP, and AuNPs:CDDP for 24 h, and the expression levels of HSPs were assessed. It was found that the protein expression of HSP40, HSP60, HSP70, and HSP90 were downregulated in A549 cells when treated with AuNPs:CDDP compared to control (Figure 5A). On the other hand, when the relative protein expression was semi-quantified using ImageJ software (Version 2.0.0, Public domain, BSD-2), it was noted that the relative protein expression of all the HSPs was significantly (*p* < 0.05) reduced with AuNPs:CDDP compared to treatment with AuNPs or CDDP alone (Figure 5B). Likewise, cell treatment with 10 µg/mL of quercetin showed similar effects to those of the combination treatment on the relative protein expression of HSPs.

### 3.6. Effects of AuNPs:CDDP on PI3K/AKT/mTOR Pathway

**Figure 6:**
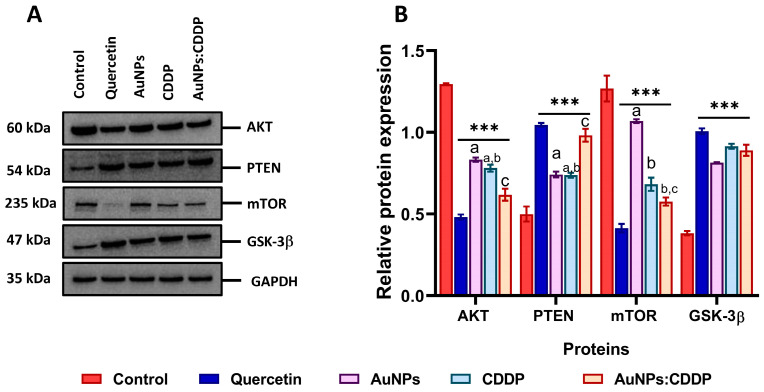
Effects of AuNPs, CDDP, and the AuNPs:CDDP combination on the expression of PI3K/AKT/mTOR pathway proteins: (**A**) protein expression of AKT, PTEN, mTOR, and GSK-3β detected by western blotting; (**B**) relative protein expression of AKT, PTEN, mTOR, and GSK-3β semi-quantified by ImageJ software (Version 2.0.0, Public domain, BSD-2). Quercetin (10 µg/mL) was used as a positive control. The plotted graphs represent the means ± SD of three independent experiments. Bars with an asterisk (*) show statistical difference (*** *p* < 0.001) compared to control. Data with different letters above the bars represent the statistically significant difference (*p* < 0.05) between the AuNPs:CDDP, CDDP, and AuNPs alone.

The combination of HSPs with AKT protein forms the HSP-AKT complex, which is essential for the functional stabilisation of the PI3K/AKT/mTOR signalling pathway [40]. To evaluate whether HSPs are involved in the phosphorylation of the AKT pathway, the expression of the AKT protein and its downstream target (such as mTOR and GSK-3β) involved in cell metabolism, survival, and progression were analysed upon treatment with AuNPs, CDDP, and the AuNPs:CDDP combination after 24 h. As shown in Figure 6A, Western blot analysis data show that the expression level of AKT and mTOR proteins were downregulated while the expression of PTEN and GSK-3β were upregulated with AuNPs:CDDP compared to AuNPs and CDDP alone. Quercetin treatment showed the same pattern in terms of the downregulation and upregulation of these proteins. When the relative protein expression was semi-quantified with ImageJ software (Version 2.0.0, Public domain, BSD-2), the AuNPs:CDDP combination showed a significant decrease (*p* < 0.05) in the relative protein expression of AKT protein compared to control, AuNPs, and CDDP alone, as shown in Figure 6B. Next, the relative protein expression of PTEN—a tumour suppressor protein that is implicated in A549 cells where it acts by interrupting the phosphorylation of AKT protein was investigated [41]. PTEN expression was found to be significantly (*p* < 0.05) upregulated with AuNPs:CDDP combination compared to AuNPs and CDDP alone. To further evaluate the effect of the combination therapy on the AKT pathway, the expression of mTOR was semi-quantified. mTOR is a downstream target of the AKT pathway that regulates protein synthesis, gene transcription, apoptosis, and autophagy [42]. The level of mTOR expression was significantly (*p* < 0.05) downregulated when treated with the AuNPs:CDDP combination. Additionally, CDDP alone also reduced (*p* < 0.001) the expression of mTOR compared to the control and AuNPs alone. Moreover, all the treatments were found to upregulate the expression of GSK-3β, a downstream target of the PI3K/AKT/mTOR pathway that is negatively regulated by AKT overactivation.

### 3.7. Effects of AuNPs:CDDP on Apoptosis-Related Proteins

**Figure 7:**
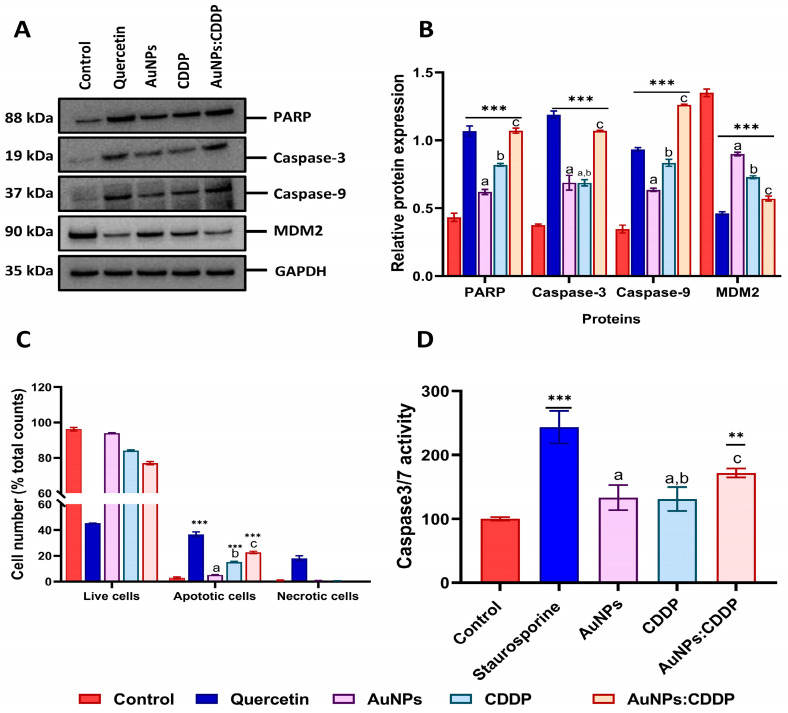
Effects of AuNPs, CDDP, and AuNPs:CDDP combination on apoptosis: (**A**) protein expression of PARP, caspase-3, caspase-9, and MDM2 detected by Western blotting; (**B**) relative protein expression of PARP, caspase-3, caspase-9, and MDM2 semi-quantified by ImageJ Software (Version 2.0.0, Public domain, BSD-2); (**C**) bar graphs analysis of apoptosis measured by flow cytometry, (**D**) caspase-3/7 activity. Quercetin (10 µg/mL) and 1 µm of Staurosporine were used as a positive controls. The plotted graphs represent the means ± SD of three independent experiments. Bars with an asterisk (*) show statistical difference (** *p* < 0.01, and *** *p* < 0.001) compared with the control. Data with different letters above the bars represent the statistically significant difference (*p* < 0.05) between the AuNPs:CDDP, CDDP, and AuNPs alone.

PI3K/AKT/mTOR pathway overactivation blocks apoptosis by preventing the release of cytochrome C from the mitochondrial inner space, inactivating the cysteine aspartyl proteases (caspases) and phosphorylating MDM2, which inhibits the tumour suppressor protein p53 [43]. To comprehend if the downregulation of the PI3K/AKT/mTOR pathway proteins upregulates apoptosis-related proteins, the expression of PARP, caspase-3, caspase-9, and MDM2 was studied following exposure to CDDP, AuNPs alone, and AuNPs:CDDP for 24 h. A total of 10 µg/mL of quercetin was used as a positive control for 24 h as it induced apoptosis by increasing the expression of caspases [44]. As shown in Figure 7A, the treatment of A549 cells with AuNPs:CDDP upregulated the expression of PARP, caspase-3, and caspase-9 and downregulated the expression of MDM2. Treatment with 10 µg/mL of quercetin showed a similar pattern in increasing the expression of PARP, caspase-3, caspase-9, and the downregulation of MDM2. When the relative protein expression was semi-quantified with ImageJ (Version 2.0.0, Public domain, BSD-2), a significant (*p* < 0.05) increase in the relative expression of PARP, caspase-3, and caspase-9 was observed with AuNPs:CDDP compared to AuNPs and CDDP alone (Figure 7B). In addition, the expression of MDM2 protein was significantly downregulated with AuNPs:CDDP combination (*p* < 0.05) compared to AuNPs and CDDP alone.

We further measured apoptosis using Annexin V/PI and caspase 3/7 assays. As shown in Figure 7C, treatment with AuNPs:CDDP augmented apoptosis by 22.65% more than that of AuNPs (5.25%) and CDDP (15.21%) alone. When compared with control, CDDP alone increased caspase 3/7 activity by 30% in A549 cells. Surprisingly, the effect was lower than that of AuNPs alone (33%). However, treatment with AuNPs:CDDP significantly increased the caspase 3/7 activity by 72% compared to the control (Figure 7D).

## 4. Discussion

In this study, the effects of combined therapy of citrate functionalised AuNPs (10 nm) with the anticancer drug CDDP were evaluated. Monodispersed AuNPs with an average size range of 8-14 nm, zeta potential of −19 ± 1.29 mV, and polydispersity index of 0.301 ± 0.01, which were characterised previously [45], were used in the current study. The use of transmission electron microscopy showed that the AuNPs were spherical with homogeneous size distribution (Figure S1 in Supplementary Materials). The effects of different concentrations of CDDP and AuNPs on cell viability were assessed using the mLDH and MTT assays. IC_50_ values were found to be 2.191 µg/mL for CDDP and 38.92 µg/mL for AuNPs. This was also highlighted by Guemei et al. [46], who found that CDDP significantly reduced A549 cell viability compared to AuNPs. In addition, AuNPs did not show any cytotoxicity to A549 cells at any of the concentrations tested (0.002–5 µg/mL) [46]. Based on our cell viability analysis, the IC_50_ value of CDDP (2.191 µg/mL) and 25 µg/mL of AuNPs were used to study the effect of the AuNPs:CDDP combination on A549 cell viability. Interestingly, the cytotoxicity assays demonstrated that the AuNPs:CDDP combination elicits greater cytotoxicity than monotherapies (CDDP and AuNPs alone). In line with our results, a previous study showed that the combination of AuNPs:CDDP had a greater potential of inhibiting the growth of HeLa (cervical), AGS (human hyperdiploid), and C6 (glioma) cell lines compared to CDDP and AuNPs alone [47]. Likewise, a combination of AuNPs:CDDP was reported to reduce the proliferation of different human ovarian cancers (A2780, OVCAR5, and SKOV3-iP) at low IC_50_ values compared to CDDP alone [48]. In addition, the cell morphology data (Figure S2 in Supplementary Materials) suggested greater cytotoxicity with the AuNPs:CDDP combination. The high cytotoxicity of the combination therapy could be mediated by the ability of AuNPs to enhance passive targeting which could improve CDDP delivery to tumours [8,49]. To gain a deeper understanding of the mechanisms of cytotoxicity, the effects of the combination therapy on oxidative stress markers were studied. The results revealed that AuNPs:CDDP induced oxidative stress by increasing ROS and reducing intracellular glutathione levels. Oxidative stress arises because of an imbalance between the production of free radicals in cells and the inability of antioxidants to neutralise them [50]. In addition, the generation of ROS induces a loss in MMP and release of mitochondrial proteins such as cytochrome C from the inner space into the cytosol which is considered to trigger caspase-dependent apoptosis [51,52]. In this work, the combination of AuNPs:CDDP was found to decrease MMP by attenuating the mitochondrial fluorescence intensity in A549 cells. Some studies suggest that the generation of ROS coupled with a decrease in MMP could lead to the activation of caspase-dependent apoptosis in A549 cancer cells [53,54,55]. Interestingly, our results for Annexin V and caspase 3/7 activity assays demonstrated similar outcomes. This suggests that apoptosis is another mechanism through which the combination therapy selectively destroyed A549 cells. In agreement with our study, Zhao et al. [8] reported that CDDP conjugation to AuNPs potentiates the antitumour effects of CDDP through apoptosis induction.

HSPs were recently recognised as a novel therapeutic target in cancer due to their roles in cell growth, differentiation, immune regulation, and proteolytic degradation [56]. These proteins are ubiquitously overexpressed in various malignant cells, including A549 cells, and their overexpression was linked to metastasis and resistance to chemotherapeutic drugs [57]. Inhibitors of HSPs are clinically limited due to their off-target toxicity and lack of in vivo data [58]. Therefore, in this work, the effect of the AuNPs:CDDP combination on HSP40, HSP60, HSP70, and HSP90 proteins was studied in A549 cells. It was observed that exposure to combination therapy downregulated the expression of all the proteins. The semi-quantification of band intensity by ImageJ software (Version 2.0.0, Public domain, BSD-2), demonstrated a significant decrease in the relative expression of the proteins (*p* < 0.05) compared with the control, CDDP, and AuNPs alone. Although the effect of AuNPs or a combination of drugs and NPs was never studied against HSPs at the time of this study, previous research showed that ibuprofen enhances the anticancer potential of CDDP by suppressing the expression of HSP70 and HSF-1 in A549 cells [59]. Studies suggested that the dysregulation of the PI3K/AKT/mTOR signalling pathway in cancer patients is mainly through HSPs and the UPS [60,61,62]. Thus, it is likely that the overexpression of HSPs mediates the expression of AKT proteins and its downstream target in A549 cells by forming the HSP-AKT complex, thereby promoting cell survival [40,63]. It was shown that the AuNPs:CDDP combination significantly downregulated the expression of AKT and mTOR proteins to a greater extent than the control. Unlike the AuNPs:CDDP combination, AuNPs alone showed no effects on the expression of either AKT or mTOR proteins. In addition, it was shown that the AuNPs:CDDP combination upregulated the expression of PTEN and GSK-3β proteins, suggesting that the combination therapy reduced the proliferation of A549 cells. This finding is consistent with other work, which showed that lowering the expression of HSPs triggers the downregulation of PI3K/AKT/mTOR pathway proteins in tumour cells [48,64].

Next, the molecular mechanisms of apoptosis were studied since both HSPs and AKT overexpression provide a protective effect against A549 apoptosis by the inhibition of apoptosome or formation of a complex with MDM2 proteins [65,66,67]. A significant upregulation of apoptotic proteins (PARP, caspase-3, and caspase-9) was observed in A549 cells when incubated with all the treatments. However, the upregulation was lower with AuNPs alone. Furthermore, the decrease in the expression of MDM2 proteins suggests the activation of apoptosis. This agrees with previous work in which an association was shown between HSP downregulations in A549 cells and the activation of apoptosis [59].

To conclude, the present study evaluated the effects of AuNPs:CDDP on HSPs, the PI3K/AKT/mTOR pathway, and apoptosis-related proteins in A549 cells. It was shown that exposure to a combination of AuNPs:CDDP modulates the expression of HSP40, HSP60, HSP70, and HSP90, resulting in the downregulation of AKT, mTOR, and MDM2 and the upregulation of PTEN, GSK-3β, PARP, caspase-3, and caspase-9. The mechanism resulting in this modulation may be associated with the increased cellular generation of ROS and a decrease in MMP. The findings also point towards further opportunities for the development of a novel therapeutic strategy that might increase the effectiveness of anticancer drugs as well as novel targets for cancer therapies.

## Data Availability

All the data presented here are available upon request from the corresponding authors.

## Supplementary Materials

The following supporting information can be downloaded at: https://www.mdpi.com/article/10.3390/pharmaceutics16030380/s1, Figure S1: Effects of AuNPs:CDDP on A549 cell viability after 24 h exposure. (A) control, (B) 20% DMSO, (C) AuNPs, (D) CDDP, (E) AuNPs:CDDP; Figure S2: Morphology and distribution of 10 nm AuNPs.
