# Supplementary material for: Modulation of Heat Shock Protein Expression in Alveolar Adenocarcinoma Cells through Gold Nanoparticles and Cisplatin Treatment
Source: Pharmaceutics. 2024 Mar 11;16(3):380. doi: 10.3390/pharmaceutics16030380 (PMC10974746; doi:10.3390/pharmaceutics16030380)
Supplement: Supplementary file 1 [file pharmaceutics-16-00380-s001.zip › pharmaceutics-2889479-supplementary.pdf]

Modulation of Heat Shock Protein Expression in Alveolar Adenocarcinoma Cells Through Gold Nanoparticles and Cisplatin

Bashiru Ibrahim[1,2]*, Taiwo Hassan Akere[1,2], Swaroop Chakraborty[2], Eugenia Valsami-Jones[2]* and Hanene Ali-Boucetta[1]*

[1] Nanomedicine, Drug Delivery & Nanotoxicology (NDDN) Lab, School of Pharmacy, College of Medical and Dental Sciences, University of Birmingham, Birmingham B15 2TT United Kingdom
[2] School of Geography, Earth and Environmental Sciences, College of Life and Environmental Sciences, University of Birmingham, Birmingham B15 2TT United Kingdom
*Correspondence: b.ibrahim@bham.ac.uk; h.aliboucetta@bham.ac.uk ; e.valsamijones@bham.ac.uk

Supplementary information

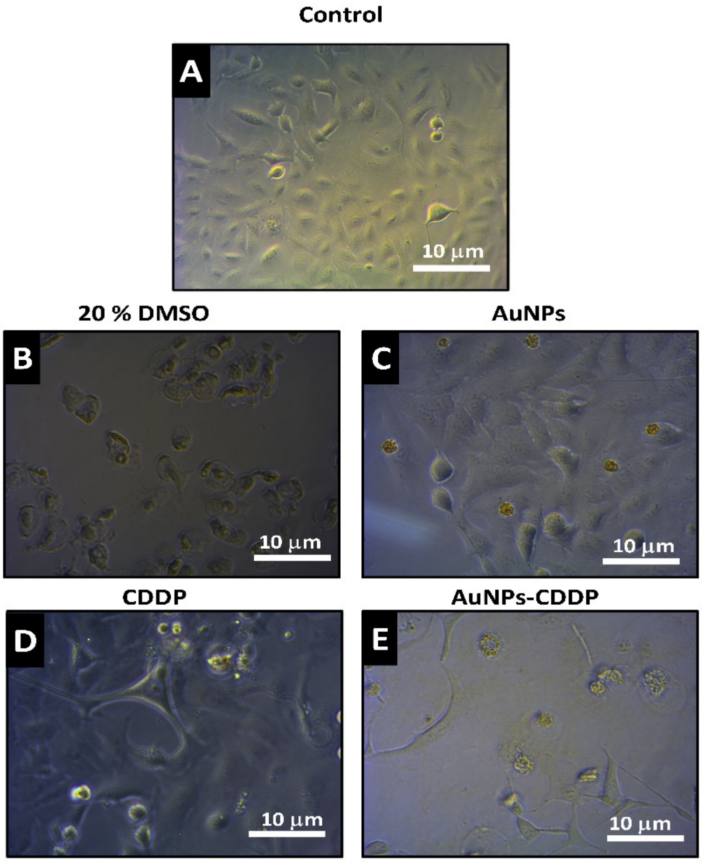

Figure S1: Effects of AuNPs-CDDP on A549 cell viability after 24 hours exposure. (A) control, (B) 20% DMSO, (C) AuNPs, (D) CDDP, (E) AuNPs:CDDP

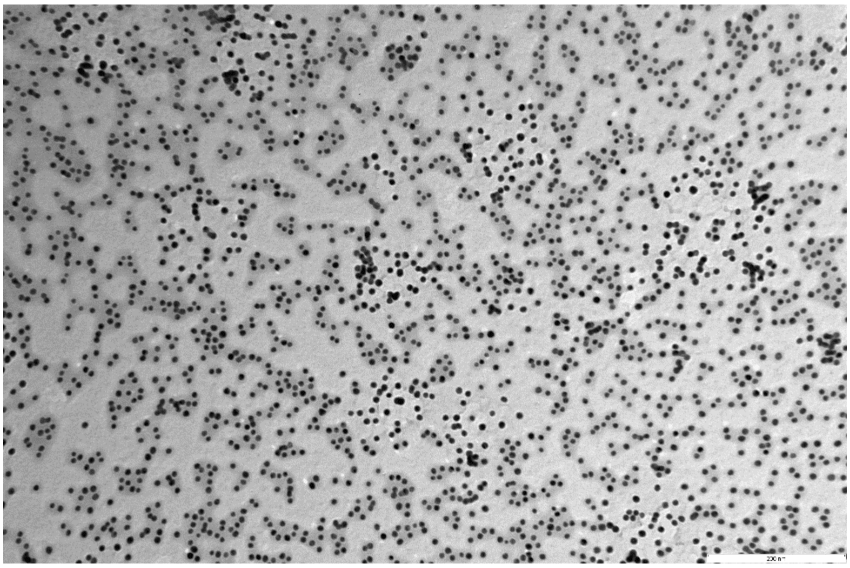

Figure S2: Morphology and distribution of 10 nm AuNPs
